# Demographical, Viro-Immunological, Clinical and Therapeutical Characteristics of HIV-Infected Patients in an “Epidemiologically Unexplored” Region of Italy (Calabria Region): the CalabrHIV Cohort

**DOI:** 10.4084/MJHID.2015.054

**Published:** 2015-10-08

**Authors:** Maria Concetta Postorino, Filippo Luciani, Carmelo Mangano, Maria Stella Carpentieri, Paolo Scerbo, Armando Priamo, Giuseppina Berardelli, Roberto Marino, Alfredo Vallone, Nicola Serrao, Vincenzo Pisani, Chiara Costa, Albano Terremoto, Giuseppe Foti, Lucio Cosco, Massimo Calderazzo, Domenico Corigliano, Preziosa Scordo, Alessio Strazzulla, Carlo Torti, The CalabrHIV Study Group

**Affiliations:** 1 Infectious Diseases Unit, *“Magna Graecia”* University, Catanzaro; 2 Infectious Diseases Unit, *“Annunziata”* Hospital, Cosenza; 3 Infectious Diseases Unit, “*Bianchi-Melacrino-Morelli”* Hospital, Reggio Calabria; 4 Infectious Diseases Unit, *“Pugliese-Ciaccio”* Hospital, Catanzaro; 5 Infectious Diseases Unit, *“Giovanni Paolo II”* Hospital, LameziaTerme, Catanzaro; 6 Infectious Diseases Unit, *“Jazzolino”* Hospital, Vibo Valentia; 7 Infectious Diseases Unit, *“San Giovanni di Dio”* Hospital, Crotone

## Abstract

**Background and Objectives:**

HIV epidemics may differ among epidemiological contexts. We aimed at constructing an HIV clinical cohort whose main epidemiological, clinical and therapeutical characteristics are described (the CalabrHIV cohort, Calabria Region, Southern Italy).

**Methods:**

The CalabrHIV Cohort includes all HIV patients on active follow-up in all infectious disease centers in the Calabria Region as at October 2014. All information was recorded in a common electronic database. Not-infectious co-morbidities (such as cardiovascular diseases, bone fractures, diabetes, renal failure and hypertension) were also studied.

**Results:**

548 patients (68% males; 59% aged <50 years) were included in the CalabrHIV cohort. Major risk factors were: sexual transmission (49%) and intravenous drug use (34%). 39% patients had HCV and/or HBV co-infection. Amongst 404 patients who had a complete clinical history, 34% were AIDS presenters and 49.3% had CD4 count ≤350/mm^3^ at HIV diagnosis. 83% patients on HAART had undetectable HIV-RNA. Hypertension was the most frequent co-morbidity (21.5%). Multimorbidity was more frequent in >50 years old patients than in <50 years old ones (30% *vs*. 6%; p<0.0001). Co-morbidity was more frequent in HCV and/or HBV co-infected than in HIV mono-infected patients (46.6% *vs*. 31.7%: p=0.0006).

**Conclusion:**

This cohort presentation study sheds light, for the first time, on HIV patients’ characteristics in the Calabria Region. We showed that HIV-infected patients with chronic hepatitis were affected by concomitant not-infectious co-morbidities more than the HIV mono-infected individuals. New HCV treatments are therefore to be implemented in the co-infected population.

## Introduction

HIV infection may give diverse clinical manifestations, due to virus-related factors, but also to host-related conditions and psycho-social peculiarities. Therefore, epidemiological and clinical features of HIV-infected patients may be different across different countries and regions of the same country, so detailed regional analyzes are very important to identify health priorities of HIV patients.

Percentages of patients with HIV infection and/or with AIDS have been underrated in the Calabria Region. Major causes may be hypothesized: stigma and marginalization due to HIV diagnosis,^1^ the psychosocial fragility of HIV population, under-reporting of new HIV diagnoses. Moreover, in the Calabria Region, incidence, prevalence and characteristics of diseases (including HIV) are affected by a massive “health migration”. Indeed, for a matter of reasons, many patients living in the Calabria Region choose to be followed in Hospitals located in the North and Centre of Italy.

In HIV patients also drug prescription can diverge from national guidelines on regional clinical practice.^2^ Moreover, there are no available data regarding premature aging and not-infective co-morbidities in HIV-infected patients from the Calabria region.^3^

So, the aim of this study was to describe baseline characteristics of HIV population in active follow-up in the Calabria Region, in order to identify health priorities of patients and create a large regional prospective cohort including all HIV-infected patients (the CalabrHIV Cohort). Main epidemiological, clinical and therapeutical characteristics have been assessed for the first time in our region. Moreover, we wanted to explore in HIV-infected patients whether or not HCV or HBV co-infection was associated with higher percentages of not-infectious co-morbidities than HIV mono-infection.

Both HIV and HCV are associated with a wide range of co-morbidities.^4^–^7^ In particular, HIV-infected patients suffer from premature aging, putting them at risk of not-infectious co-morbidities at younger ages than the general population.^3^,^4^ Indeed, high levels of predictive biomarkers of inflammation typical of the great elderly people were found in young people with HIV infection.^3^ Previous cohort studies showed a greater risk of diabetes mellitus, acute myocardial infarction and cerebrovascular events in HIV-infected patients than in HIV-negative ones.^8^–^12^ It is currently not demonstrated whether HCV or HBV co-infection may accelerate premature aging in HIV-infected patients, increasing rates of cardiovascular, metabolic and renal diseases in these subjects. In conclusion, the reasons leading to the study were:

1. To present a cohort profile of the CalabrHIV study group.
2. To focus on clinical characteristics of the enrolled patients, mainly the risk of comorbidities.

## Materials and Methods

All the infectious diseases centers in the Calabria Region (Catanzaro, Vibo Valentia, Reggio Calabria, Cosenza, and Crotone) have merged their data to create a regional observational prospective cohort, so called CalabrHIV Cohort. Patients characteristics were collected in a common electronic database containing all epidemiological, demographic, virological, immunological, clinical and therapeutic information. Latest follow-up was available in October 2014.

Not-infectious comorbidities diagnosed until October 2014 were also recorded: cardiovascular diseases (defined as acute myocardial infarction, stroke, transient ischemic attack, angina pectoris, coronary bypass, angioplasty, chronic occlusive arterial disease), hypertension (defined as blood pressure ≥140/90 mmHg or antihypertensive therapy), diabetes (fasting serum glucose ≥126 mg/dl or anti-diabetes therapy), renal failure (eGFR ≤60 ml/min measured with CKD-EPI formula)^13^ and bone fractures. Multimorbidity was defined as ≥2 not-infectious co-morbidities occurring in the same patient. For the analysis of not-infectious co-morbidities, patients were divided into four age groups: aged ≤40, between 41 and 50 years, between 51 and 60 years and >60 years.

Data were analyzed using common statistical descriptive procedures (with statistical significance: p≤0.05).

## Results

Five hundred forty-eight patients (68% males; 59% aged <50 years) on active follow-up as at October 2014 were included in the CalabrHIV Cohort. Table 1 reports the main patients demographic, epidemiological and clinical characteristics. Major risk factors for HIV acquisition were sexual transmission (49%) and intravenous drug use (34%). About 40% patients had HCV and/or HBV co-infection.

### AIDS and late presentation

Although a good virological response was ongoing (73% had undetectable HIV-RNA), the immunological status of patients, with a CD4 nadir <200/mm3, was still compromised. Only 42% of these patients had actual CD4 count >500/mm3). 404/548 patients had available data about AIDS at HIV diagnosis, of these 34% were AIDS presenters, and 49.3% had CD4 count ≤350/mm3.

### HAART prescription

90% patients were on HAART and, among these, 83% had undetectable HIV-RNA. A huge diversity in HAART prescription was noticed: 92% first-line HAART included ≥3 different drugs. Only 5% first line prescriptions included <3 antiretroviral drugs but, amongst the currently ongoing regimens, 15% included <3 drugs as simplification regimens. 49% patients actually in mono-/ dual- therapy *vs.* 40% patients actually in HAART had a nadir CD4 <200/mm3. Actual HIV RNA was <50 copies/ml in 81% patients receiving mono-/ dual- therapy and in 83% of patients on HAART. The actual CD4 T cell count was >500/mm3 in 60% patients receiving mono-/ dual- therapy and in 62% patients on HAART.

### Study of not infectious co-morbidities

**Figure 1:**
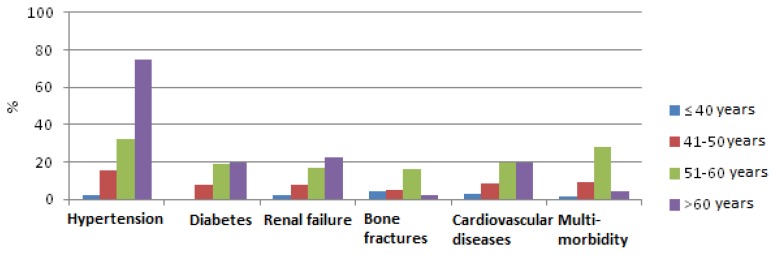
Not-infectious co-morbidities (%) by age classes

**Figure 2:**
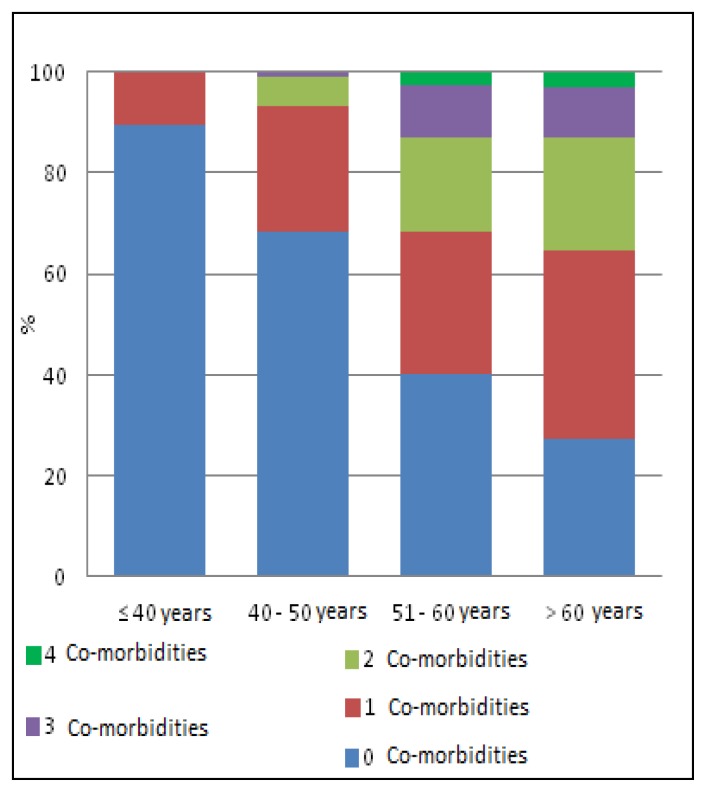
Multi-morbidity (% by age classes) in CalabrHIV cohort.

Median value of not infectious comorbidities per patient was 0.58 (range 0–4). 21.5% patients had at least one co-morbidity. None of the patients had five, not infectious comorbidities. Hypertension was the most frequent disease (21.5% patients) followed by cardiovascular diseases (11.5%), renal failure (10%) and diabetes (10%) (Figure 1). Multimorbidity was more frequently found higher in >50 years old patients than in <50 years old (30% *vs*. 6%; p<0.0001) (Figure 2).

Patients were ranked into two groups: HIV mono-infected patients (61%) and HIV patients co-infected with HCV and/or HBV (39%).

Table 2 shows main epidemiological, clinical and demographic characteristics of HIV mono-infected patients and HIV patients coinfected with HCV and/or HBV. Co-infected patients were significantly older [mean age 49 years (SD 8.7) *vs*. 46 years (SD 12.7); p=0.0003] and more frequently had AIDS than mono-infected ones (39% *vs*. 21%; p<0.0001). Although not statistically significant, nadir and actual CD4 T cell values were lower in co-infected patients than in mono-infected ones.

**Figure 3:**
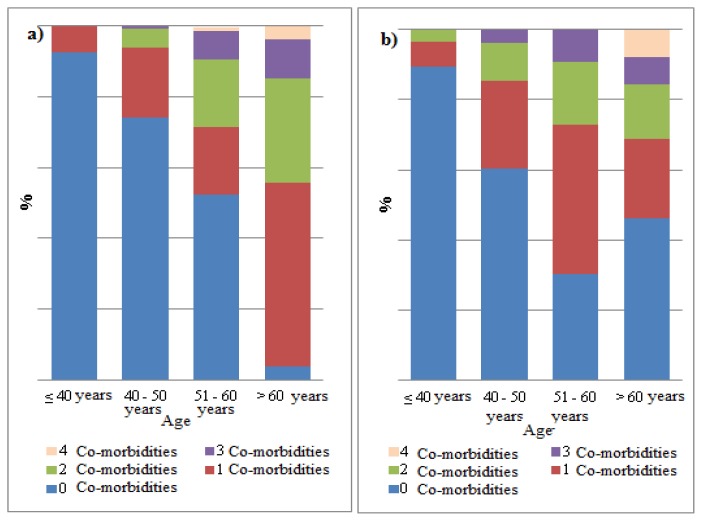
**a)** % Multi-morbidity in HIV mono infected patients; **b)** % Multi-morbidity in HIV patients co-infected with viral hepatitis

Multimorbidity rate was higher in patients aged ≥50 years with HCV and/or HBV co-infection than in HIV-mono-infected (70% *vs.* 46%; p=0.0037). A increased significance in the difference due to multimorbidity was found in age groups starting from 40 years (39.5% *vs*. 26%; p=0.0293), although difference was even inverse at >60 years of age (54% *vs*. 92%; p=0.026) (Figure 3a–b). There was a difference in bone fractures rates between HIV mono-infected patients and patients with HCV and/or HBV co-infection at a borderline significance value (5.2% *vs.* 9%; p=0.07).

## Discussion

This paper describes, for the first time, the main epidemiological and clinical features of HIV patients in the Calabria Region. This large number of patients with HIV infection, included in our study, suggests that the HIV/AIDS epidemics in the Calabria Region is more important than currently believed. More than 500 patients were on active follow-up, notwithstanding that the last estimates of National Institute of Health reported the lowest incidence of new HIV diagnoses among Italian regions in Calabria (0.2/100,000 inhabitants in 2012, 1.4/100,000 in 2013).^14^ As previously suggested, underreporting, under-testing due to the fear of stigma and marginalization, and the health migration phenomenon to areas of the North/Centre of Italy may be some causes of this bias.^1^

Main demographic and clinical characteristics of CalabrHIV Cohort may be compared with other national cohorts. In particular, patients of CalabrHIV Cohort are older (patients age was mainly up to 40 years old) than patients belonging to the Italian MASTER Cohort (mean age 38.5 years old).^15^

This datum may be due to selection bias since patients of older age may be those less prone to migration. Alternatively, it may reflect a later diagnosis (i.e., HIV infection is discovered later in life). However, our data are consistent with national estimates; that reported a progressive increase in mean age of patients diagnosed with HIV/AIDS in Italy.^14^

Percentages of late presenters in the CalabrHIV Cohort were similar to those reported recently in Europe and Italy.^16^–^19^ About one third HIV patients in Europe were late presenters.^19^ Data from the Italian AIDS Registry from 1982 to 2011 showed a progressively increased proportion of AIDS diagnoses in patients aged >49 years in the latest years.^18^ Older patients with AIDS were more frequently males, late testers and diagnosed with AIDS in more recent years than younger patients.^18^ Rates of late presentation may vary by country, by nationality and by transmission patterns. As reported in a recent international study, rates of AIDS diagnosis within three months from HIV diagnosis in Italy was 14.5%.^17^ In Italy people, presenting late acquired infection more frequently by heterosexual contact, whereas, in other countries, greater rates of late presenters were reported among intravenous drug users.^17^ Late presentation was associated with a higher rates of AIDS and mortality, in particular during the first year after HIV diagnosis.^19^ Moreover, patients presented late showed a greater risk of HAART not-adherence, drug toxicity, disease progression and death with respect to patients who presented earlier.^20^

National guidelines may be interpreted and applied differently in different regional contexts. National HIV/AIDS guidelines do not recommend mono-/ dual-therapy as standard regimens.^21^ At present, a valuable percentage of patients in the CalabrHIV cohort is treated with <3 drugs (15%) and their viro-immunological profiles are similar to that of patients on HAART. Viro-immunological results in our patients are consistent with those recently published.^22^

HIV infection accelerates the normal process of aging.^3^ Previous studies showed a higher prevalence of not infectious comorbidities (such as diabetes mellitus) and a greater relative risk of acute myocardial infarction in HIV-infected patients compared with HIV-negative ones.^8^–^10^ Also, risk of cerebrovascular diseases was higher in patients with HIV infection.^12^ At the same time, previous cohort studies do not explain the possible current association between HIV infection and other risk factors of not infectious co-morbidities (such as smoking, obesity, or family related risk).^11^,^12^,^23^

Prevalence of not-infectious comorbidities and multimorbidity rate in CalabrHIV cohort were similar to those reported by *Guaraldi et al*.^4^ However, in that study, patients already diagnosed with lipodystrophy and/or metabolic diseases were included, whereas, in the CalabrHIV Cohort, HIV population may be more similar to the general.

It is important to highlight that comorbidities rate was significantly greater in HIV patients coinfected with HCV and/or HBV than in HIV mono-infected patients (46.6% *vs*. 31.7%: p=0.0006); this datum was not published yet. Also multimorbidity rate was higher in co-infected patients than in HIV-mono-infected ones. This effect was not driven by the oldest subjects since an increasing significance was found with the increase in age starting from 40 years. Hepatitis viruses co-infection may modify the natural history of HIV infections, further accelerating premature aging.^24^–^26^ Interestingly, difference in co-morbidity rates was even inverse (54% *vs*. 92%; p=0.026) at >60 years of age in our cohort, probably because of age-related risk diluted the impact of HCV and/or HBV co-infection in the elderly. In conclusion, our results suggest that a greater attention should be given to HIV co-infected patients, particularly to those of younger age. Since eradication of HCV was associated not only with prevention of liver-related morbidity and mortality^27^ but also with prevention of not infectious events, treatment of HCV with the new drugs^28^,^29^ should be implemented.

Aims of CalabrHIV Study Group are to continue prospective follow-up and patients’ recruitment. Prevention and early detection of not infectious comorbidities are important, in particular in the younger ones. Treatment of HCV should be extended to patients infected with HIV with the aim of improving both liver and general conditions of patients.

## Figures and Tables

**Table 1:** Patient characteristics.

	CalabrHIV (n=548)
**Qualitative variables**	**%**
**Gender**	
- Males	68
**Age classes**	
- ≤40 years	24
- 41–50 years	35
- 51–60 years	33
- >60 years	8
**Risk factors for HIV acquisition**	
- Intravenous drug use	34
- Sexual transmission	49
- Other/unknown	17
**Actual CD4+/mm^3^ classes**	
- <350	20.5
- 350–500	17.3
- >500	60.2
- Unknown	2
**HIV-RNA (copies/ml)**	
- Detectable	26
- Undetectable	74
**HAART prescription**	90
**Co-infections**	
- HCV Ab+	32
- Chronic HBsAg carriers	5
- Chronic HBsAg carriers/HCV Ab+	2
**Quantitative variables**	
Mean age (SD)	47 (10)
Nadir CD4+/mm^3^ mean (SD)	243 (204)
Actual CD4+/mm^3^ mean (SD)	637 (392)
Median HIV RNA copies/ml (range)	33 (20–160,000)

Undetectable HIV-RNA: <34 copies/ml

SD: standard deviation

**Table 2:** Patients characteristics (HIV positive patients *vs.* HIV patients co-infected with viral hepatitis).

	HIV (n=338)	HIV/HBV/HCV (n=210)	P value
**Qualitative variables (%)**			
**Gender**			
- Males	64	77	<0.0001*
- Females	36	23	
**Age classes**			
- ≤40 years	30	14	<0.0001*
- 41–50 years	34	38	0.4209
- 51–60 years	26	44	<0.0001*
- >60 years	10	4	0.0121
**Risk factors**			
- Intravenous drug use	16	69	<0.0001*
- Sexual transmission	75	23	<0.0001*
- Other/unknown	9	8	0.9677
**Actual CD4+/mm^3^ classes**			
- <350	17.5	27.5	0.0047*
- 350 – 500	24.2	18.5	0.0791
- >500	58.3	54	0.1866
**HIV-RNA (copies/ml)**			
- Undetectable	73	75	0.7949
- Detectable	27	25	
**AIDS**	21	39	<0.0001*
**Multi-morbidity**			
Age ≤40 years	7.5	10	0.4281
Age 41–50 years	26	39.5	0.0293
Age 51–60 years	46	70	0.0037
Age >60 years	92	54	0.0026
**Bone fractures (%)**	5.2	9	0.0701
**Quantitative variables**			
Mean age (SD)	46 (12.4)	49 (8.7)	0.0003*
Mean CD4+/mm^3^ nadir (SD)	431 (188)	231 (172)	0.1897
Mean actual CD4+/mm^3^ (SD)	579 (390)	649 (451)	0.2260

Undetectable HIV-RNA: <34 copies/ml

SD: standard deviation

(*) indicates variables with statistical significance (p≤0.05)
